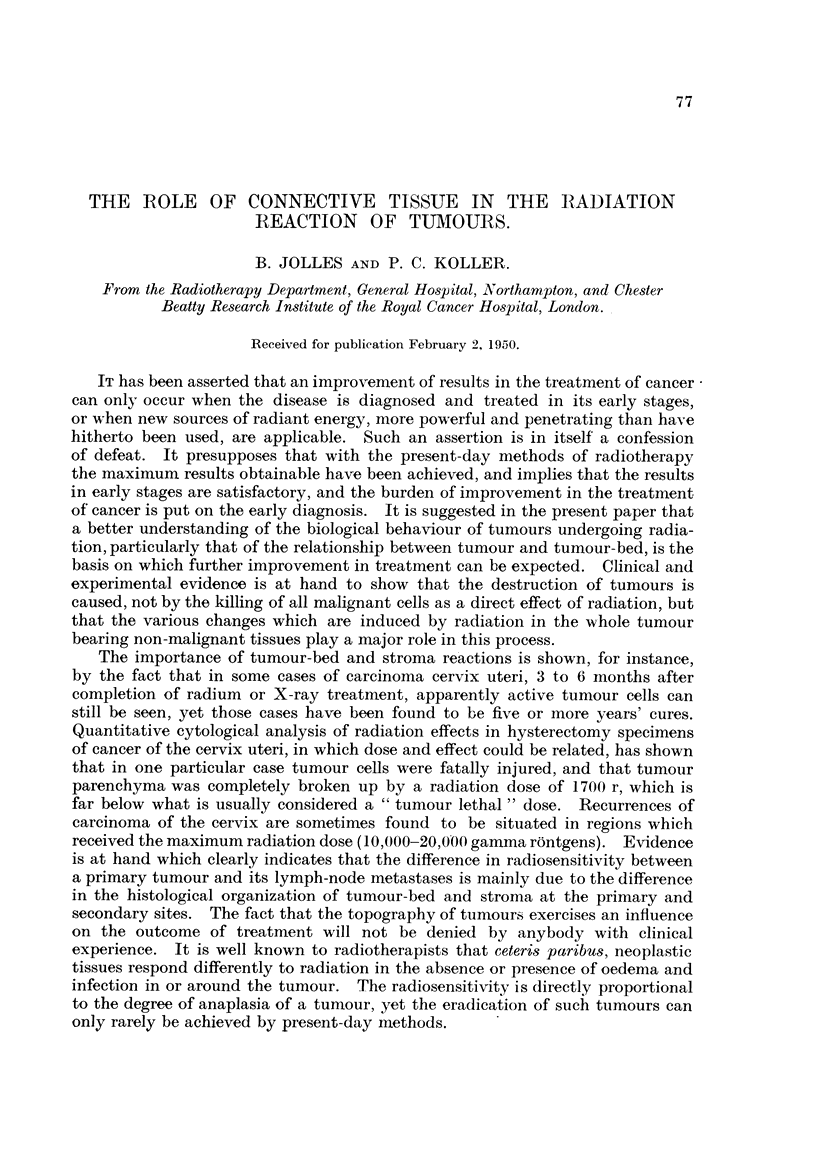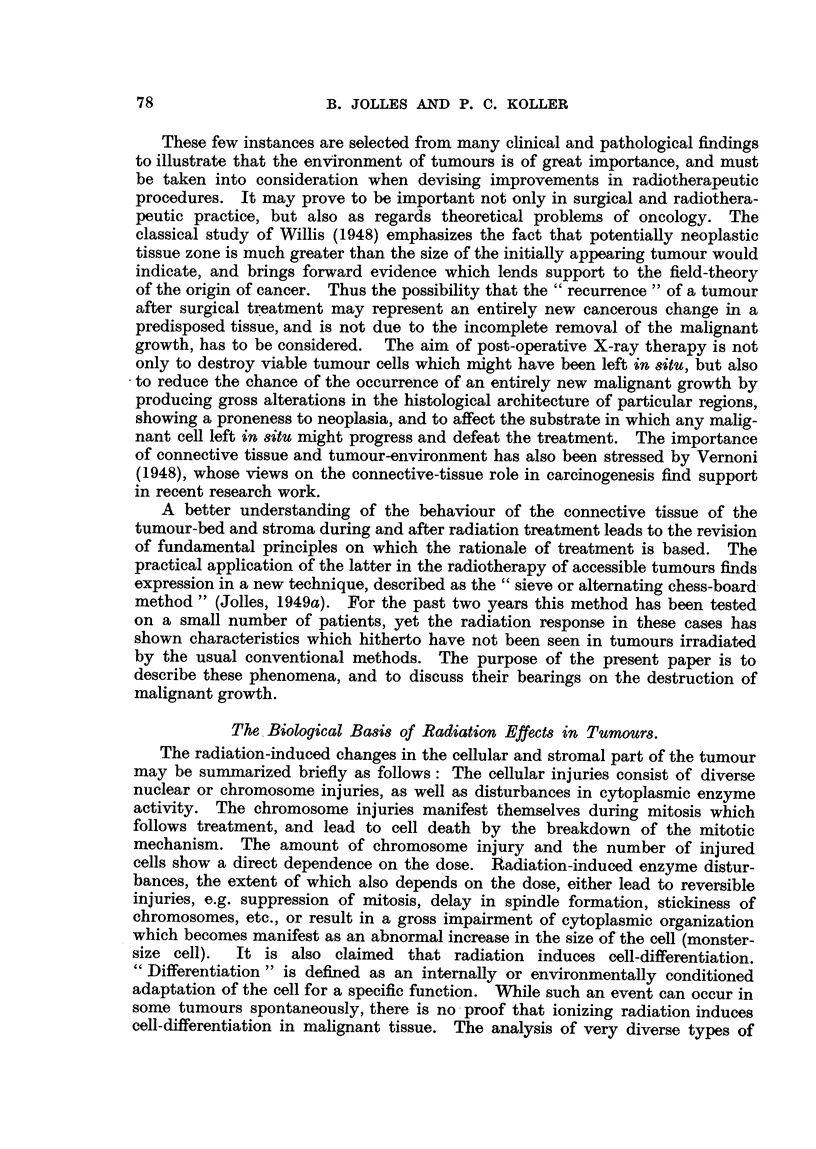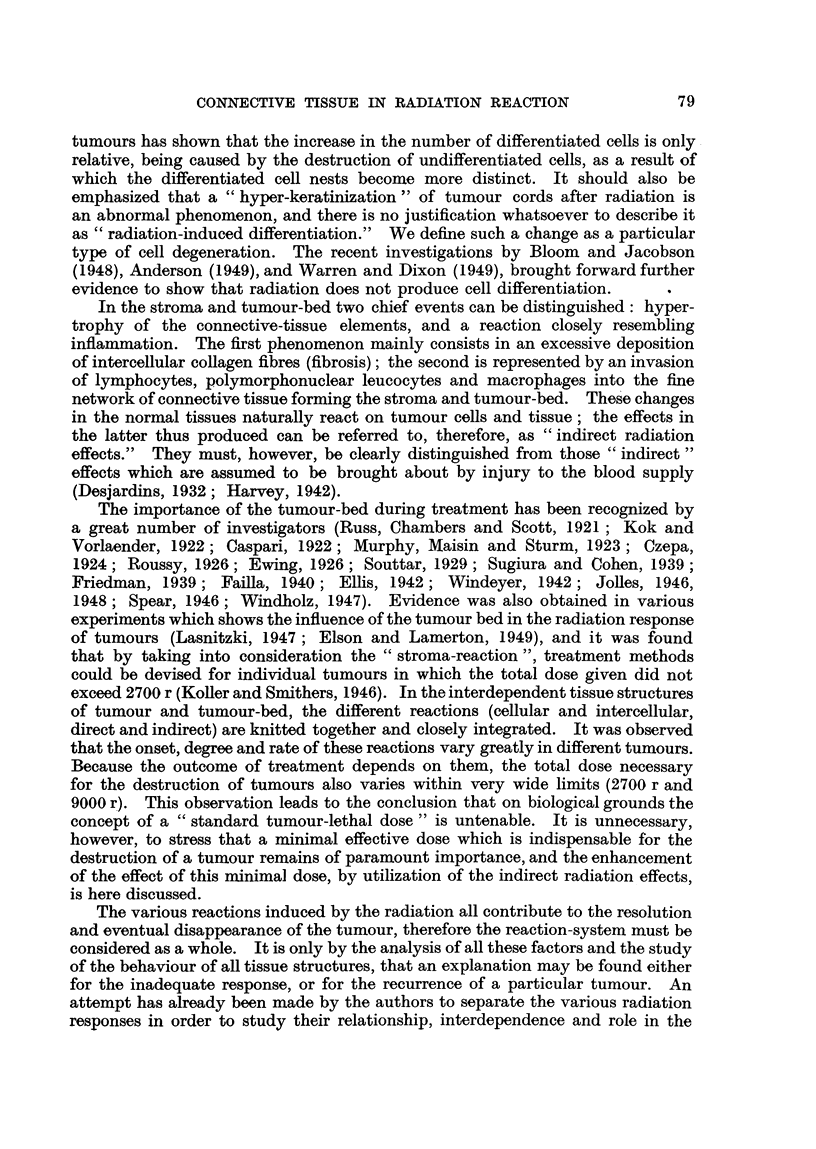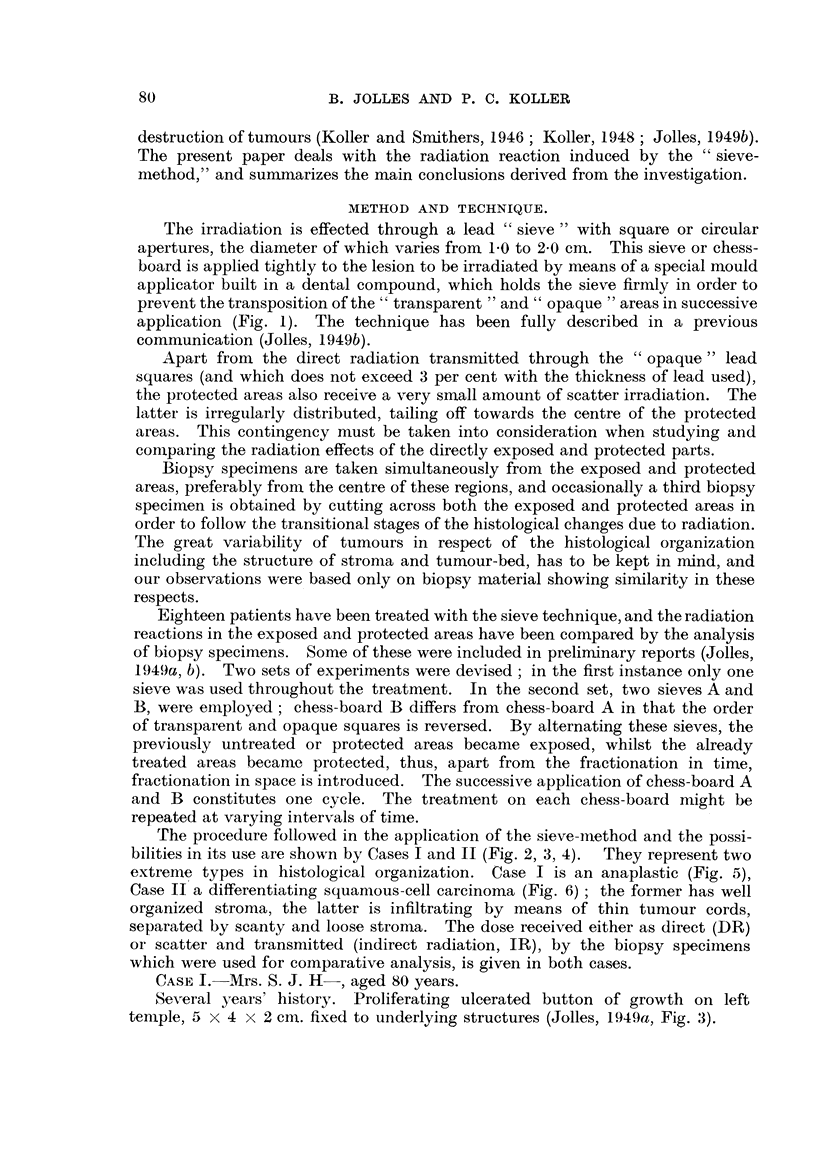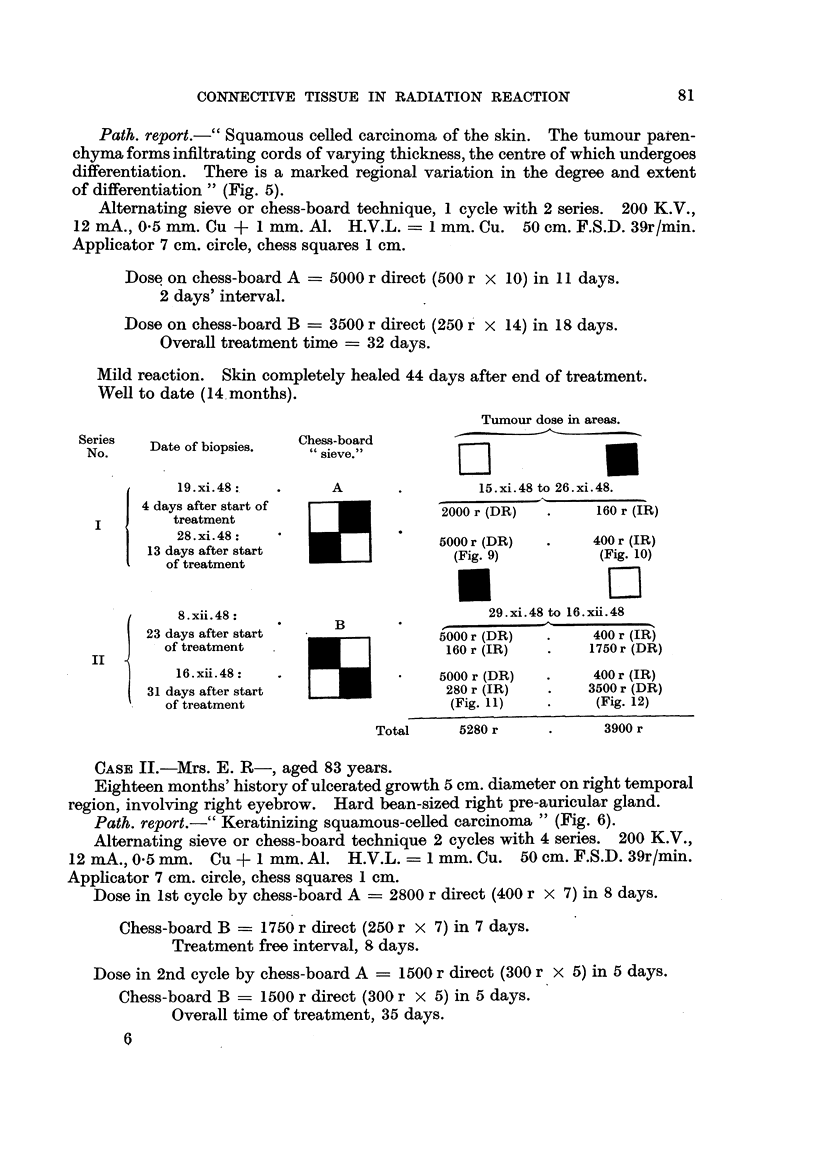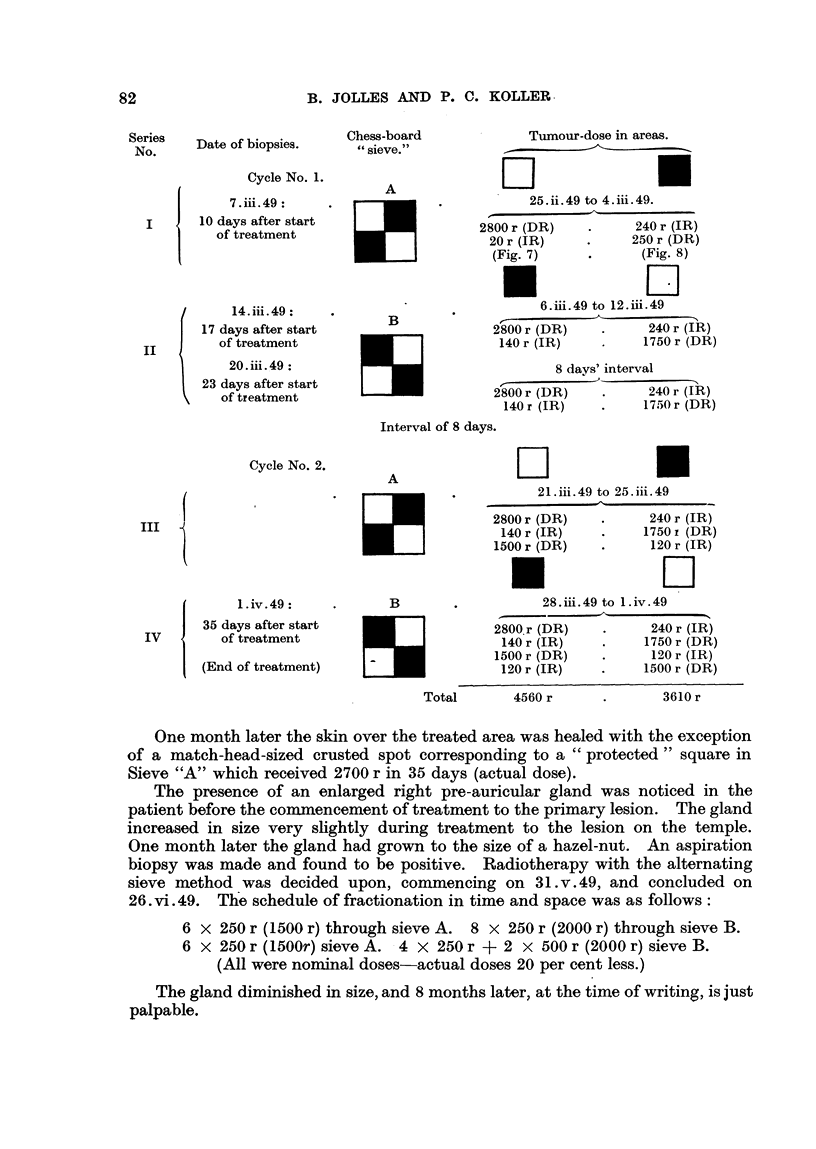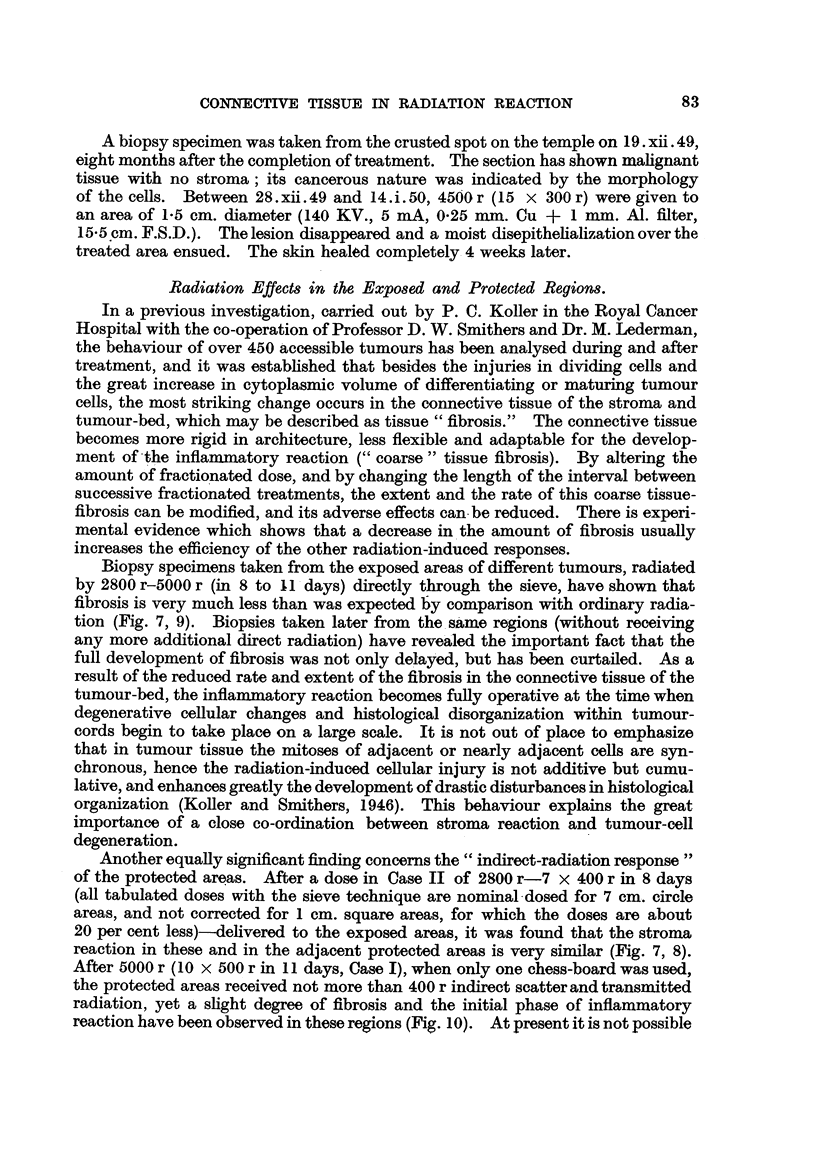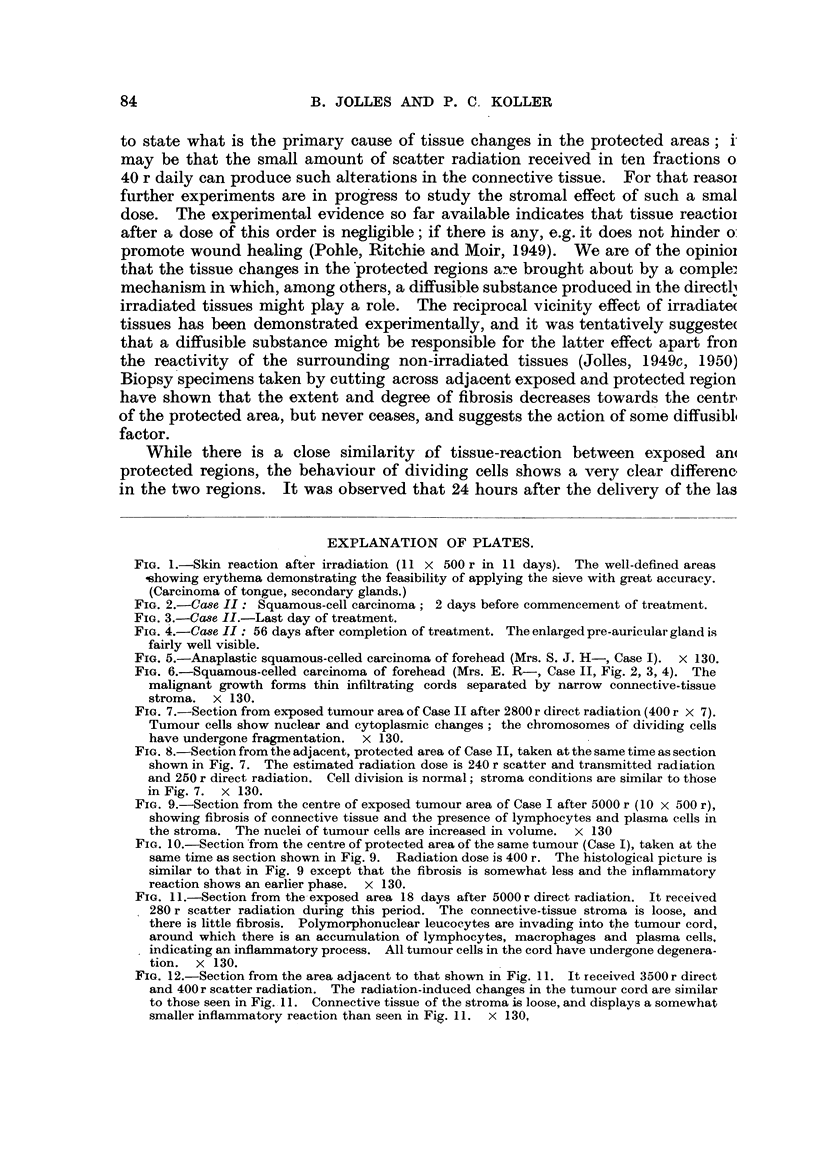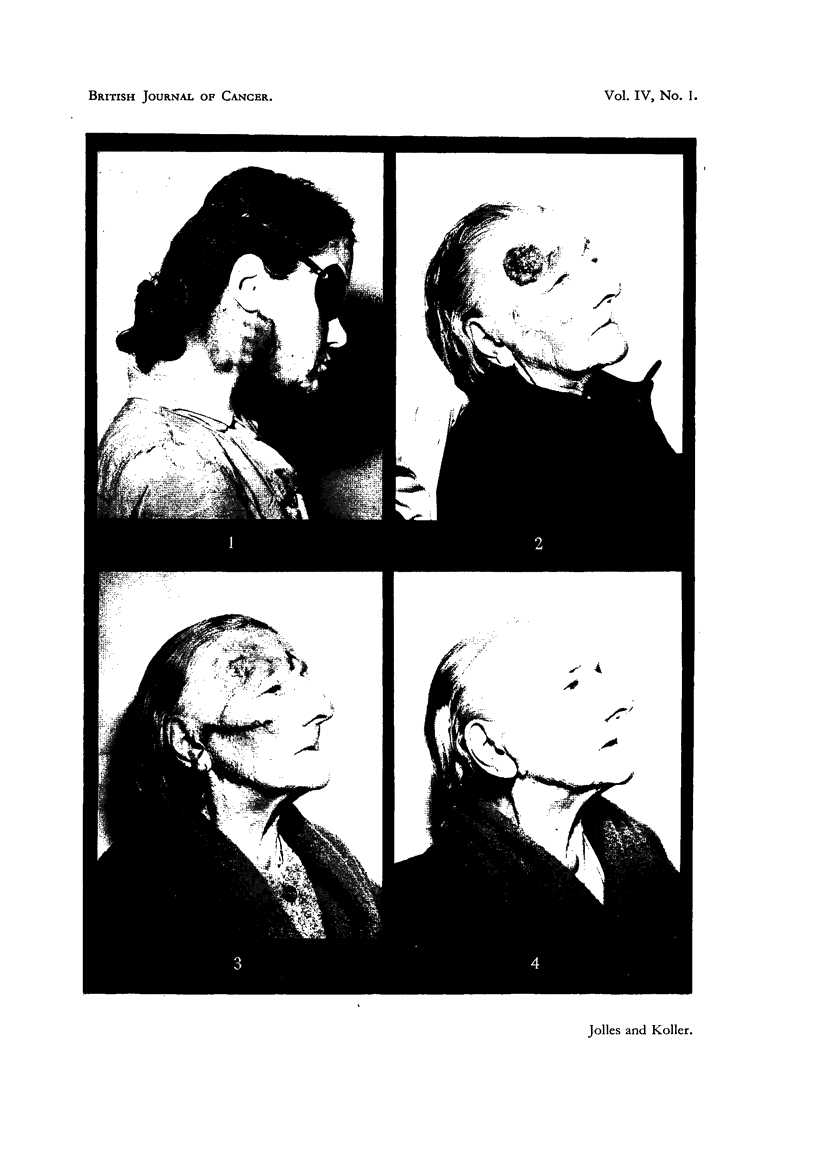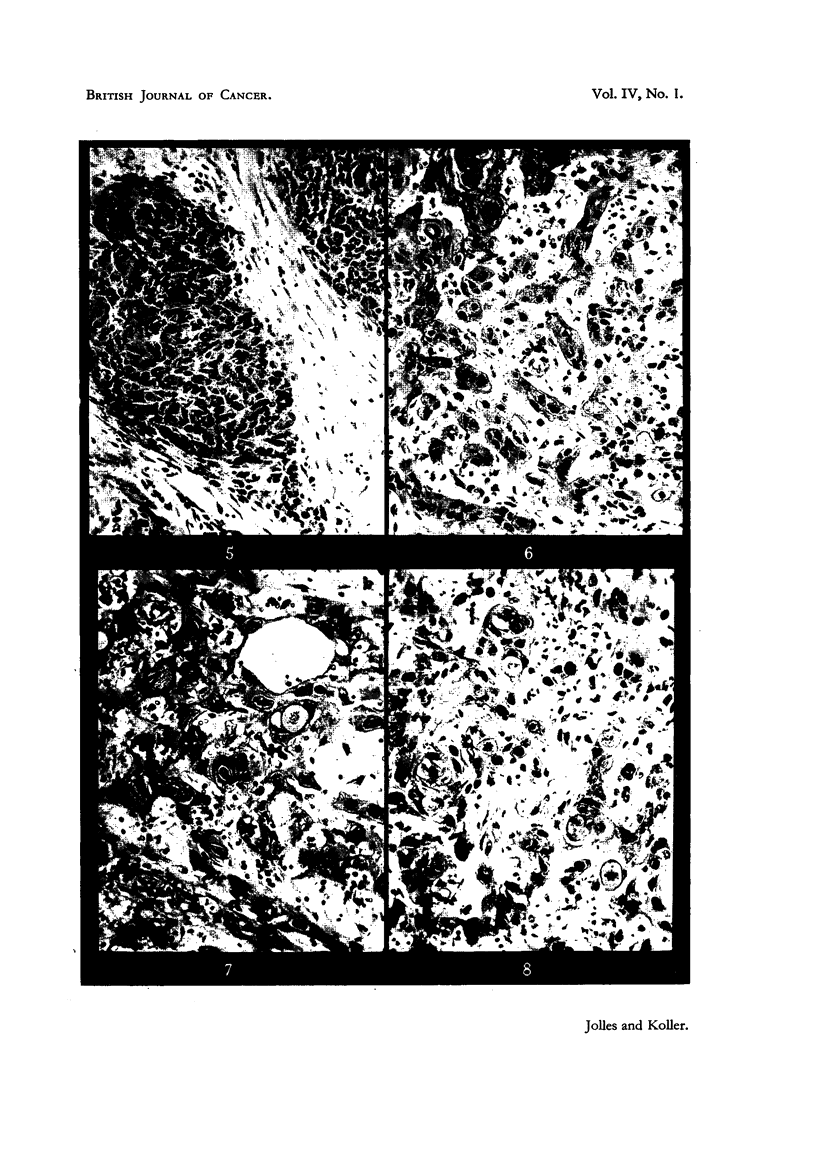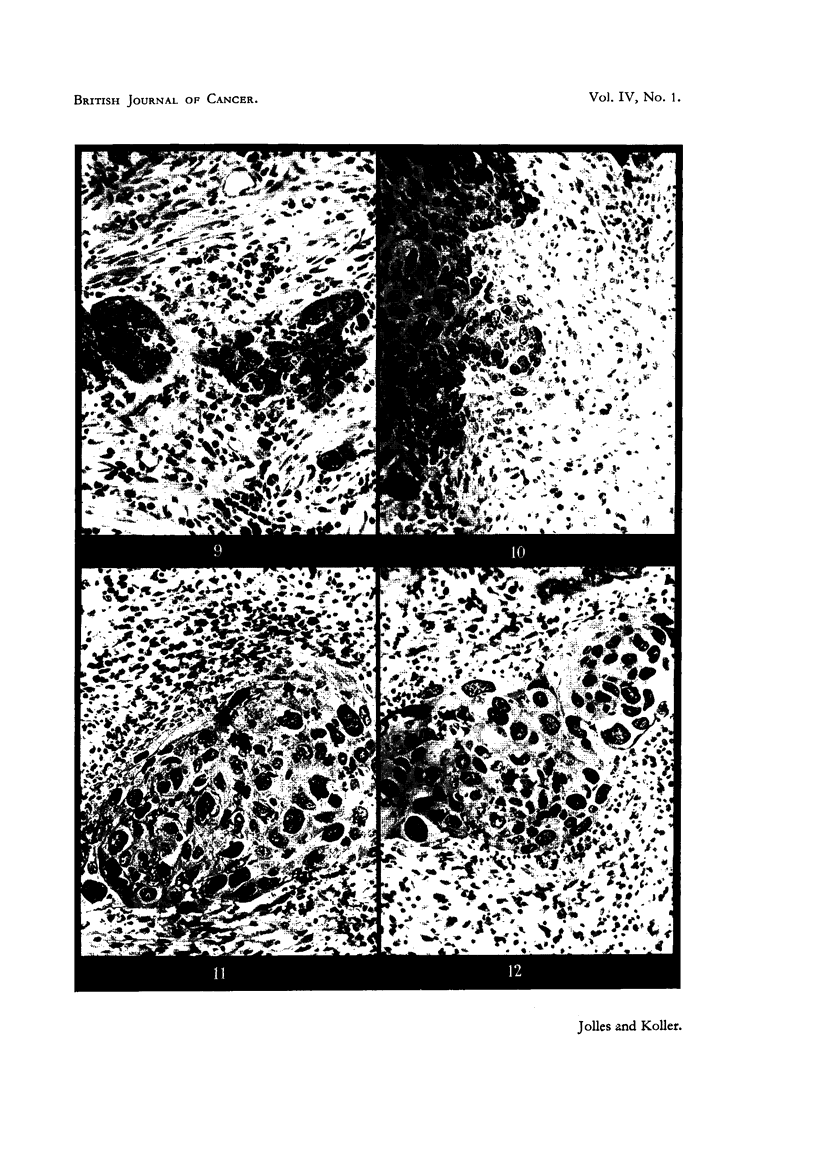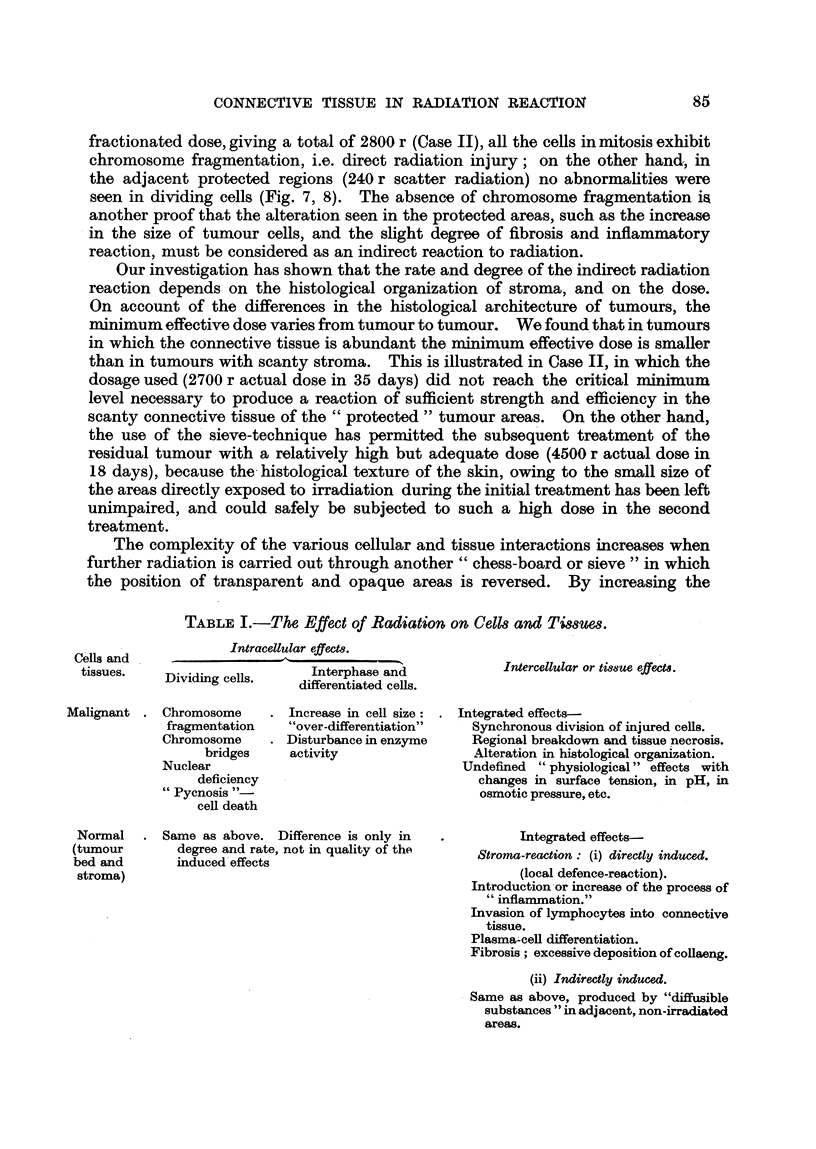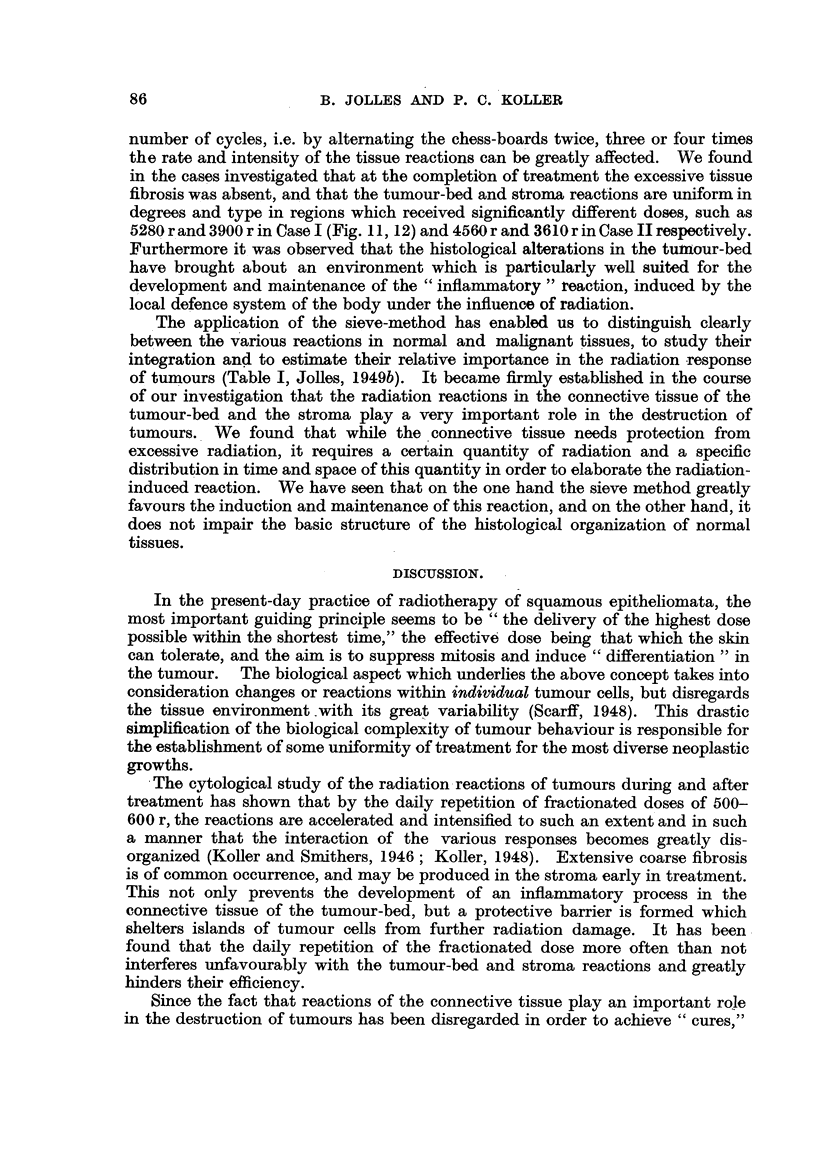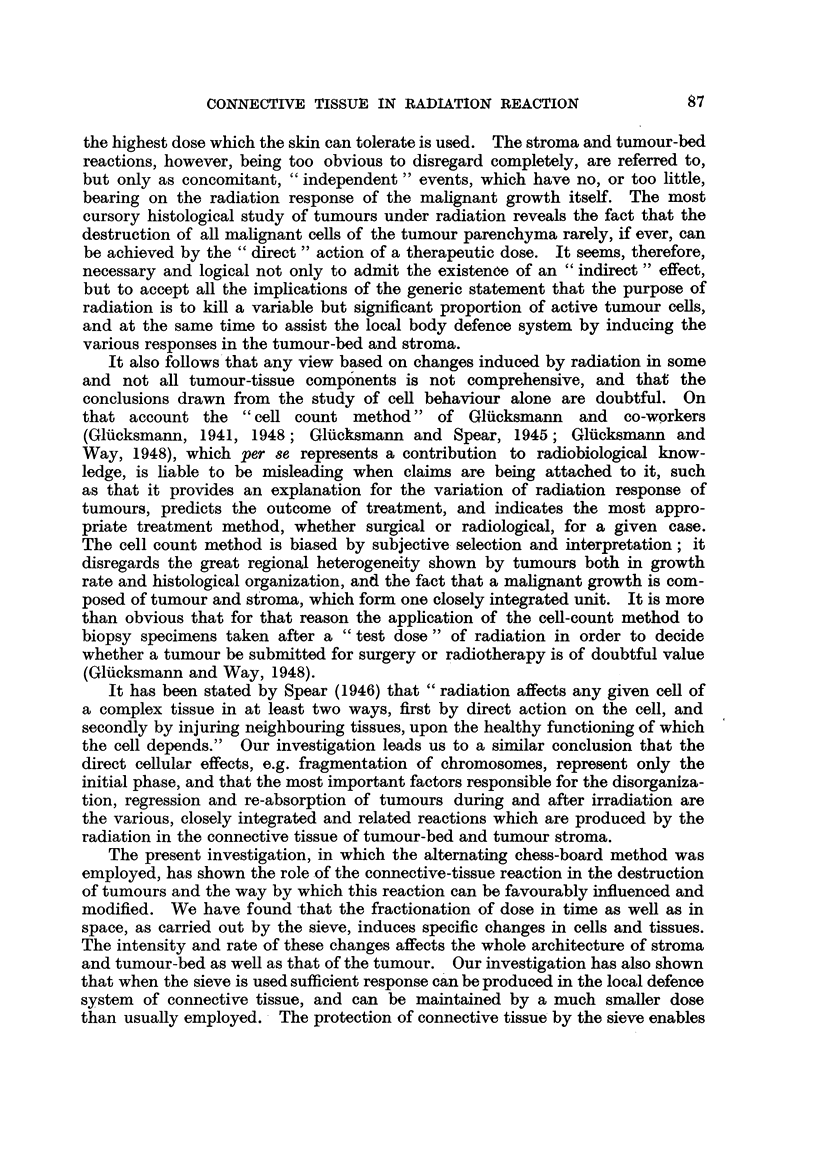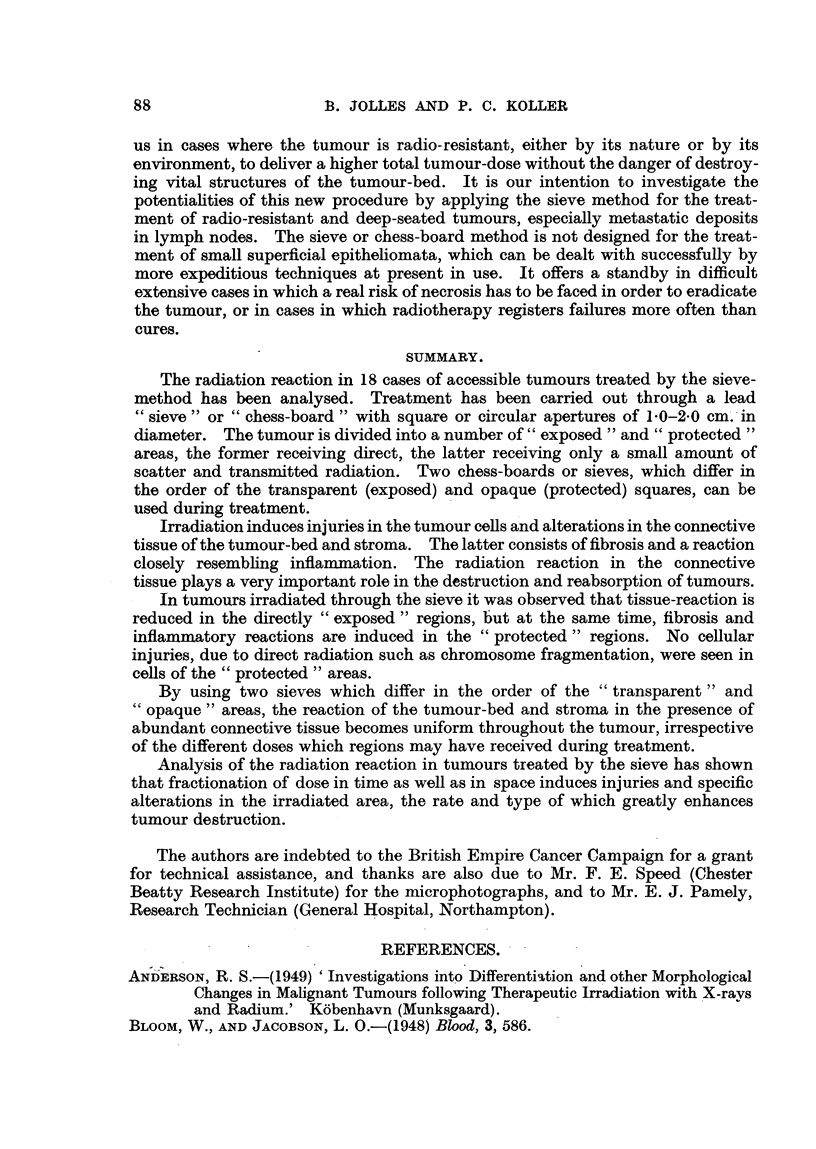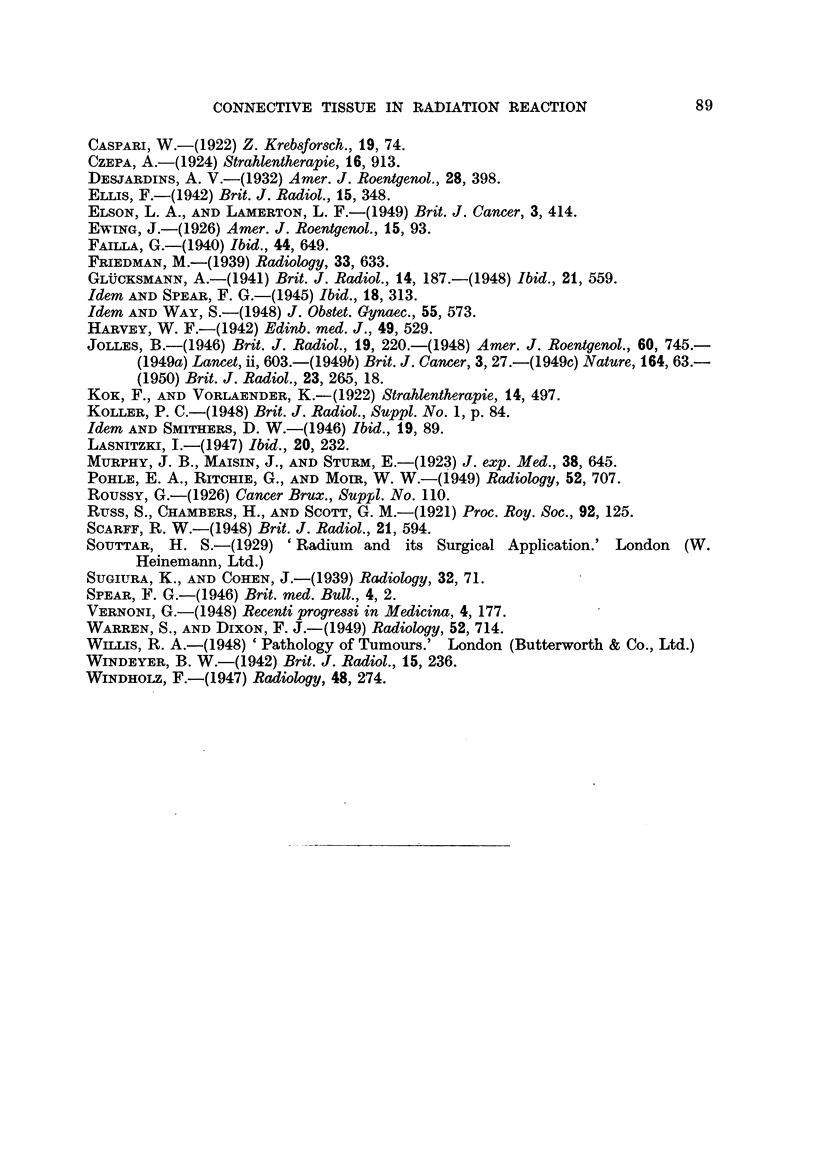# The Role of Connective Tissue in the Radiation Reaction of Tumours

**DOI:** 10.1038/bjc.1950.6

**Published:** 1950-03

**Authors:** B. Jolles, P. C. Koller

## Abstract

**Images:**


### THE ROLE OF CONNECTIVE TISSUE IN THE RADIATION REACTION OF TUMOURS.

B. JOLLES AND P. C. KOLLER.

*From the Radiotherapy Department, General Hospital, Northampton, and Chester Beatty Research Institute of the Royal Cancer Hospital, London.*

Received for publication February 2, 1950.

IT has been asserted that an improvement of results in the treatment of cancer can only occur when the disease is diagnosed and treated in its early stages, or when new sources of radiant energy, more powerful and penetrating than have hitherto been used, are applicable. Such an assertion is in itself a confession of defeat. It presupposes that with the present-day methods of radiotherapy the maximum results obtainable have been achieved, and implies that the results in early stages are satisfactory, and the burden of improvement in the treatment of cancer is put on the early diagnosis. It is suggested in the present paper that a better understanding of the biological behaviour of tumours undergoing radiation, particularly that of the relationship between tumour and tumour-bed, is the basis on which further improvement in treatment can be expected. Clinical and experimental evidence is at hand to show that the destruction of tumours is caused, not by the killing of all malignant cells as a direct effect of radiation, but that the various changes which are induced by radiation in the whole tumour bearing non-malignant tissues play a major role in this process.

The importance of tumour-bed and stroma reactions is shown, for instance, by the fact that in some cases of carcinoma cervix uteri, 3 to 6 months after completion of radium or X-ray treatment, apparently active tumour cells can still be seen, yet those cases have been found to be five or more years' cures. Quantitative cytological analysis of radiation effects in hysterectomy specimens of cancer of the cervix uteri, in which dose and effect could be related, has shown that in one particular case tumour cells were fatally injured, and that tumour parenchyma was completely broken up by a radiation dose of 1700 r, which is far below what is usually considered a " tumour lethal " dose. Recurrences of carcinoma of the cervix are sometimes found to be situated in regions which received the maximum radiation dose (10,000–20,000 gamma röntgens). Evidence is at hand which clearly indicates that the difference in radiosensitivity between a primary tumour and its lymph-node metastases is mainly due to the difference in the histological organization of tumour-bed and stroma at the primary and secondary sites. The fact that the topography of tumours exercises an influence on the outcome of treatment will not be denied by anybody with clinical experience. It is well known to radiotherapists that *ceteris paribus*, neoplastic tissues respond differently to radiation in the absence or presence of oedema and infection in or around the tumour. The radiosensitivity is directly proportional to the degree of anaplasia of a tumour, yet the eradication of such tumours can only rarely be achieved by present-day methods.

These few instances are selected from many clinical and pathological findings to illustrate that the environment of tumours is of great importance, and must be taken into consideration when devising improvements in radiotherapeutic procedures. It may prove to be important not only in surgical and radiotherapeutic practice, but also as regards theoretical problems of oncology. The classical study of Willis (1948) emphasizes the fact that potentially neoplastic tissue zone is much greater than the size of the initially appearing tumour would indicate, and brings forward evidence which lends support to the field-theory of the origin of cancer. Thus the possibility that the " recurrence " of a tumour after surgical treatment may represent an entirely new cancerous change in a predisposed tissue, and is not due to the incomplete removal of the malignant growth, has to be considered. The aim of post-operative X-ray therapy is not only to destroy viable tumour cells which might have been left *in situ*, but also to reduce the chance of the occurrence of an entirely new malignant growth by producing gross alterations in the histological architecture of particular regions, showing a proneness to neoplasia, and to affect the substrate in which any malignant cell left *in situ* might progress and defeat the treatment. The importance of connective tissue and tumour-environment has also been stressed by Vernoni (1948), whose views on the connective-tissue role in carcinogenesis find support in recent research work.

A better understanding of the behaviour of the connective tissue of the tumour-bed and stroma during and after radiation treatment leads to the revision of fundamental principles on which the rationale of treatment is based. The practical application of the latter in the radiotherapy of accessible tumours finds expression in a new technique, described as the " sieve or alternating chess-board method " (Jolles, 1949*a*). For the past two years this method has been tested on a small number of patients, yet the radiation response in these cases has shown characteristics which hitherto have not been seen in tumours irradiated by the usual conventional methods. The purpose of the present paper is to describe these phenomena, and to discuss their bearings on the destruction of malignant growth.

### *The Biological Basis of Radiation Effects in Tumours.*

The radiation-induced changes in the cellular and stromal part of the tumour may be summarized briefly as follows : The cellular injuries consist of diverse nuclear or chromosome injuries, as well as disturbances in cytoplasmic enzyme activity. The chromosome injuries manifest themselves during mitosis which follows treatment, and lead to cell death by the breakdown of the mitotic mechanism. The amount of chromosome injury and the number of injured cells show a direct dependence on the dose. Radiation-induced enzyme disturbances, the extent of which also depends on the dose, either lead to reversible injuries, e.g. suppression of mitosis, delay in spindle formation, stickiness of chromosomes, etc., or result in a gross impairment of cytoplasmic organization which becomes manifest as an abnormal increase in the size of the cell (monster-size cell). It is also claimed that radiation induces cell-differentiation. " Differentiation " is defined as an internally or environmentally conditioned adaptation of the cell for a specific function. While such an event can occur in some tumours spontaneously, there is no proof that ionizing radiation induces cell-differentiation in malignant tissue. The analysis of very diverse types of tumours has shown that the increase in the number of differentiated cells is only relative, being caused by the destruction of undifferentiated cells, as a result of which the differentiated cell nests become more distinct. It should also be emphasized that a " hyper-keratinization " of tumour cords after radiation is an abnormal phenomenon, and there is no justification whatsoever to describe it as " radiation-induced differentiation." We define such a change as a particular type of cell degeneration. The recent investigations by Bloom and Jacobson (1948), Anderson (1949), and Warren and Dixon (1949), brought forward further evidence to show that radiation does not produce cell differentiation.

In the stroma and tumour-bed two chief events can be distinguished : hypertrophy of the connective-tissue elements, and a reaction closely resembling inflammation. The first phenomenon mainly consists in an excessive deposition of intercellular collagen fibres (fibrosis) ; the second is represented by an invasion of lymphocytes, polymorphonuclear leucocytes and macrophages into the fine network of connective tissue forming the stroma and tumour-bed. These changes in the normal tissues naturally react on tumour cells and tissue ; the effects in the latter thus produced can be referred to, therefore, as " indirect radiation effects." They must, however, be clearly distinguished from those " indirect " effects which are assumed to be brought about by injury to the blood supply (Desjardins, 1932 ; Harvey, 1942).

The importance of the tumour-bed during treatment has been recognized by a great number of investigators (Russ, Chambers and Scott, 1921 ; Kok and Vorlaender, 1922 ; Caspari, 1922 ; Murphy, Maisin and Sturm, 1923 ; Czepa, 1924 ; Roussy, 1926 ; Ewing, 1926 ; Souttar, 1929 ; Sugiura and Cohen, 1939 ; Friedman, 1939 ; Failla, 1940 ; Ellis, 1942 ; Windeyer, 1942 ; Jolles, 1946, 1948 ; Spear, 1946 ; Windholz, 1947). Evidence was also obtained in various experiments which shows the influence of the tumour bed in the radiation response of tumours (Lasnitzki, 1947 ; Elson and Lamerton, 1949), and it was found that by taking into consideration the " stroma-reaction ", treatment methods could be devised for individual tumours in which the total dose given did not exceed 2700 r (Koller and Smithers, 1946). In the interdependent tissue structures of tumour and tumour-bed, the different reactions (cellular and intercellular, direct and indirect) are knitted together and closely integrated. It was observed that the onset, degree and rate of these reactions vary greatly in different tumours. Because the outcome of treatment depends on them, the total dose necessary for the destruction of tumours also varies within very wide limits (2700 r and 9000 r). This observation leads to the conclusion that on biological grounds the concept of a " standard tumour-lethal dose " is untenable. It is unnecessary, however, to stress that a minimal effective dose which is indispensable for the destruction of a tumour remains of paramount importance, and the enhancement of the effect of this minimal dose, by utilization of the indirect radiation effects, is here discussed.

The various reactions induced by the radiation all contribute to the resolution and eventual disappearance of the tumour, therefore the reaction-system must be considered as a whole. It is only by the analysis of all these factors and the study of the behaviour of all tissue structures, that an explanation may be found either for the inadequate response, or for the recurrence of a particular tumour. An attempt has already been made by the authors to separate the various radiation responses in order to study their relationship, interdependence and role in the destruction of tumours (Koller and Smithers, 1946 ; Koller, 1948 ; Jolles, 1949*b*). The present paper deals with the radiation reaction induced by the " sieve-method," and summarizes the main conclusions derived from the investigation.

### METHOD AND TECHNIQUE.

The irradiation is effected through a lead " sieve " with square or circular apertures, the diameter of which varies from 1·0 to 2·0 cm. This sieve or chess-board is applied tightly to the lesion to be irradiated by means of a special mould applicator built in a dental compound, which holds the sieve firmly in order to prevent the transposition of the " transparent " and " opaque " areas in successive application (Fig. 1). The technique has been fully described in a previous communication (Jolles, 1949*b*).

Apart from the direct radiation transmitted through the " opaque " lead squares (and which does not exceed 3 per cent with the thickness of lead used), the protected areas also receive a very small amount of scatter irradiation. The latter is irregularly distributed, tailing off towards the centre of the protected areas. This contingency must be taken into consideration when studying and comparing the radiation effects of the directly exposed and protected parts.

Biopsy specimens are taken simultaneously from the exposed and protected areas, preferably from the centre of these regions, and occasionally a third biopsy specimen is obtained by cutting across both the exposed and protected areas in order to follow the transitional stages of the histological changes due to radiation. The great variability of tumours in respect of the histological organization including the structure of stroma and tumour-bed, has to be kept in mind, and our observations were based only on biopsy material showing similarity in these respects.

Eighteen patients have been treated with the sieve technique, and the radiation reactions in the exposed and protected areas have been compared by the analysis of biopsy specimens. Some of these were included in preliminary reports (Jolles, 1949*a*, *b*). Two sets of experiments were devised ; in the first instance only one sieve was used throughout the treatment. In the second set, two sieves A and B, were employed ; chess-board B differs from chess-board A in that the order of transparent and opaque squares is reversed. By alternating these sieves, the previously untreated or protected areas became exposed, whilst the already treated areas became protected, thus, apart from the fractionation in time, fractionation in space is introduced. The successive application of chess-board A and B constitutes one cycle. The treatment on each chess-board might be repeated at varying intervals of time.

The procedure followed in the application of the sieve-method and the possibilities in its use are shown by Cases I and II (Fig. 2, 3, 4). They represent two extreme types in histological organization. Case I is an anaplastic (Fig. 5), Case II a differentiating squamous-cell carcinoma (Fig. 6) ; the former has well organized stroma, the latter is infiltrating by means of thin tumour cords, separated by scanty and loose stroma. The dose received either as direct (DR) or scatter and transmitted (indirect radiation, IR), by the biopsy specimens which were used for comparative analysis, is given in both cases.

CASE I.—Mrs. S. J. H—, aged 80 years.

Several years' history. Proliferating ulcerated button of growth on left temple, 5 × 4 × 2 cm. fixed to underlying structures (Jolles, 1949*a*, Fig. 3).

*Path. report.*—" Squamous celled carcinoma of the skin. The tumour parenchyma forms infiltrating cords of varying thickness, the centre of which undergoes differentiation. There is a marked regional variation in the degree and extent of differentiation " (Fig. 5).

Alternating sieve or chess-board technique, 1 cycle with 2 series. 200 K.V., 12 mA., 0·5 mm. Cu + 1 mm. Al. H.V.L. = 1 mm. Cu. 50 cm. F.S.D. 39r/min. Applicator 7 cm. circle, chess squares 1 cm.

Dose on chess-board A = 5000 r direct (500 r × 10) in 11 days.
2 days' interval.

Dose on chess-board B = 3500 r direct (250 r × 14) in 18 days.
Overall treatment time = 32 days.

Mild reaction. Skin completely healed 44 days after end of treatment. Well to date (14 months).

| Series No. | Date of biopsies. | Chess-board " sieve." | Tumour dose in areas.<br> |  |
|---|---|---|---|---|
| I | 19.xi.48 :<br>4 days after start of treatment | A<br> | 15.xi.48 to 26.xi.48.<br>2000 r (DR) | 160 r (IR) |
| | 28.xi.48 :<br>13 days after start of treatment | | 5000 r (DR)<br>(Fig. 9) | 400 r (IR)<br>(Fig. 10) |
| | | |  |  |
| II | 8.xii.48 :<br>23 days after start of treatment | B<br> | 29.xi.48 to 16.xii.48<br>5000 r (DR)<br>160 r (IR) | 400 r (IR)<br>1750 r (DR) |
| | 16.xii.48 :<br>31 days after start of treatment | | 5000 r (DR)<br>280 r (IR)<br>(Fig. 11) | 400 r (IR)<br>3500 r (DR)<br>(Fig. 12) |
| | | Total | 5280 r | 3900 r |

CASE II.—Mrs. E. R—, aged 83 years.

Eighteen months' history of ulcerated growth 5 cm. diameter on right temporal region, involving right eyebrow. Hard bean-sized right pre-auricular gland.

*Path. report.*—" Keratinizing squamous-celled carcinoma " (Fig. 6).

Alternating sieve or chess-board technique 2 cycles with 4 series. 200 K.V., 12 mA., 0·5 mm. Cu + 1 mm. Al. H.V.L. = 1 mm. Cu. 50 cm. F.S.D. 39r/min. Applicator 7 cm. circle, chess squares 1 cm.

Dose in 1st cycle by chess-board A = 2800 r direct (400 r × 7) in 8 days.

Chess-board B = 1750 r direct (250 r × 7) in 7 days.
Treatment free interval, 8 days.

Dose in 2nd cycle by chess-board A = 1500 r direct (300 r × 5) in 5 days.
Chess-board B = 1500 r direct (300 r × 5) in 5 days.
Overall time of treatment, 35 days.

| Series No. | Date of biopsies. | Chess-board "sieve." | Tumour-dose in areas. | |
|---|---|---|---|---|
| | Cycle No. 1. | | □ | ■ |
| I | 7.iii.49 : 10 days after start of treatment | A  | 25.ii.49 to 4.iii.49. | |
| | | | 2800 r (DR) 20 r (IR) (Fig. 7) | 240 r (IR) 250 r (DR) (Fig. 8) |
| | | | ■ | □ |
| II | 14.iii.49 : 17 days after start of treatment | B  | 6.iii.49 to 12.iii.49 | |
| | | | 2800 r (DR) 140 r (IR) | 240 r (IR) 1750 r (DR) |
| | 20.iii.49 : 23 days after start of treatment | | 8 days' interval | |
| | | | 2800 r (DR) 140 r (IR) | 240 r (IR) 1750 r (DR) |
| | | Interval of 8 days. | | |
| | Cycle No. 2. | | □ | ■ |
| III | | A  | 21.iii.49 to 25.iii.49 | |
| | | | 2800 r (DR) 140 r (IR) 1500 r (DR) | 240 r (IR) 1750 r (DR) 120 r (IR) |
| | | | ■ | □ |
| IV | 1.iv.49 : 35 days after start of treatment (End of treatment) | B  | 28.iii.49 to 1.iv.49 | |
| | | | 2800 r (DR) 140 r (IR) 1500 r (DR) 120 r (IR) | 240 r (IR) 1750 r (DR) 120 r (IR) 1500 r (DR) |
| | | Total | 4560 r | 3610 r |

One month later the skin over the treated area was healed with the exception of a match-head-sized crusted spot corresponding to a " protected " square in Sieve "A" which received 2700 r in 35 days (actual dose).

The presence of an enlarged right pre-auricular gland was noticed in the patient before the commencement of treatment to the primary lesion. The gland increased in size very slightly during treatment to the lesion on the temple. One month later the gland had grown to the size of a hazel-nut. An aspiration biopsy was made and found to be positive. Radiotherapy with the alternating sieve method was decided upon, commencing on 31.v.49, and concluded on 26.vi.49. The schedule of fractionation in time and space was as follows :

6 × 250 r (1500 r) through sieve A. 8 × 250 r (2000 r) through sieve B.

6 × 250 r (1500*r*) sieve A. 4 × 250 r + 2 × 500 r (2000 r) sieve B.

(All were nominal doses—actual doses 20 per cent less.)

The gland diminished in size, and 8 months later, at the time of writing, is just palpable.

A biopsy specimen was taken from the crusted spot on the temple on 19.xii.49, eight months after the completion of treatment. The section has shown malignant tissue with no stroma ; its cancerous nature was indicated by the morphology of the cells. Between 28.xii.49 and 14.i.50, 4500 r (15 × 300 r) were given to an area of 1·5 cm. diameter (140 KV., 5 mA, 0·25 mm. Cu + 1 mm. Al. filter, 15·5 cm. F.S.D.). The lesion disappeared and a moist disepithelialization over the treated area ensued. The skin healed completely 4 weeks later.

*Radiation Effects in the Exposed and Protected Regions.*

In a previous investigation, carried out by P. C. Koller in the Royal Cancer Hospital with the co-operation of Professor D. W. Smithers and Dr. M. Lederman, the behaviour of over 450 accessible tumours has been analysed during and after treatment, and it was established that besides the injuries in dividing cells and the great increase in cytoplasmic volume of differentiating or maturing tumour cells, the most striking change occurs in the connective tissue of the stroma and tumour-bed, which may be described as tissue " fibrosis." The connective tissue becomes more rigid in architecture, less flexible and adaptable for the development of the inflammatory reaction (" coarse " tissue fibrosis). By altering the amount of fractionated dose, and by changing the length of the interval between successive fractionated treatments, the extent and the rate of this coarse tissue-fibrosis can be modified, and its adverse effects can be reduced. There is experimental evidence which shows that a decrease in the amount of fibrosis usually increases the efficiency of the other radiation-induced responses.

Biopsy specimens taken from the exposed areas of different tumours, radiated by 2800 r–5000 r (in 8 to 11 days) directly through the sieve, have shown that fibrosis is very much less than was expected by comparison with ordinary radiation (Fig. 7, 9). Biopsies taken later from the same regions (without receiving any more additional direct radiation) have revealed the important fact that the full development of fibrosis was not only delayed, but has been curtailed. As a result of the reduced rate and extent of the fibrosis in the connective tissue of the tumour-bed, the inflammatory reaction becomes fully operative at the time when degenerative cellular changes and histological disorganization within tumour-cords begin to take place on a large scale. It is not out of place to emphasize that in tumour tissue the mitoses of adjacent or nearly adjacent cells are synchronous, hence the radiation-induced cellular injury is not additive but cumulative, and enhances greatly the development of drastic disturbances in histological organization (Koller and Smithers, 1946). This behaviour explains the great importance of a close co-ordination between stroma reaction and tumour-cell degeneration.

Another equally significant finding concerns the " indirect-radiation response " of the protected areas. After a dose in Case II of 2800 r—7 × 400 r in 8 days (all tabulated doses with the sieve technique are nominal dosed for 7 cm. circle areas, and not corrected for 1 cm. square areas, for which the doses are about 20 per cent less)—delivered to the exposed areas, it was found that the stroma reaction in these and in the adjacent protected areas is very similar (Fig. 7, 8). After 5000 r (10 × 500 r in 11 days, Case I), when only one chess-board was used, the protected areas received not more than 400 r indirect scatter and transmitted radiation, yet a slight degree of fibrosis and the initial phase of inflammatory reaction have been observed in these regions (Fig. 10). At present it is not possible to state what is the primary cause of tissue changes in the protected areas; i may be that the small amount of scatter radiation received in ten fractions o 40 r daily can produce such alterations in the connective tissue. For that reasoı further experiments are in progress to study the stromal effect of such a smal dose. The experimental evidence so far available indicates that tissue reactioı after a dose of this order is negligible; if there is any, e.g. it does not hinder o promote wound healing (Pohle, Ritchie and Moir, 1949). We are of the opinioı that the tissue changes in the protected regions are brought about by a comple mechanism in which, among others, a diffusible substance produced in the directl irradiated tissues might play a role. The reciprocal vicinity effect of irradiate tissues has been demonstrated experimentally, and it was tentatively suggeste that a diffusible substance might be responsible for the latter effect apart fron the reactivity of the surrounding non-irradiated tissues (Jolles, 1949*c*, 1950) Biopsy specimens taken by cutting across adjacent exposed and protected region have shown that the extent and degree of fibrosis decreases towards the centr of the protected area, but never ceases, and suggests the action of some diffusibl factor.

While there is a close similarity of tissue-reaction between exposed an protected regions, the behaviour of dividing cells shows a very clear differenc in the two regions. It was observed that 24 hours after the delivery of the las

---

EXPLANATION OF PLATES.

FIG. 1.—Skin reaction after irradiation (11 × 500 r in 11 days). The well-defined areas showing erythema demonstrating the feasibility of applying the sieve with great accuracy. (Carcinoma of tongue, secondary glands.)

FIG. 2.—*Case II*: Squamous-cell carcinoma; 2 days before commencement of treatment.

FIG. 3.—*Case II.*—Last day of treatment.

FIG. 4.—*Case II*: 56 days after completion of treatment. The enlarged pre-auricular gland is fairly well visible.

FIG. 5.—Anaplastic squamous-celled carcinoma of forehead (Mrs. S. J. H—, Case I). × 130.

FIG. 6.—Squamous-celled carcinoma of forehead (Mrs. E. R—, Case II, Fig. 2, 3, 4). The malignant growth forms thin infiltrating cords separated by narrow connective-tissue stroma. × 130.

FIG. 7.—Section from exposed tumour area of Case II after 2800 r direct radiation (400 r × 7). Tumour cells show nuclear and cytoplasmic changes; the chromosomes of dividing cells have undergone fragmentation. × 130.

FIG. 8.—Section from the adjacent, protected area of Case II, taken at the same time as section shown in Fig. 7. The estimated radiation dose is 240 r scatter and transmitted radiation and 250 r direct radiation. Cell division is normal; stroma conditions are similar to those in Fig. 7. × 130.

FIG. 9.—Section from the centre of exposed tumour area of Case I after 5000 r (10 × 500 r), showing fibrosis of connective tissue and the presence of lymphocytes and plasma cells in the stroma. The nuclei of tumour cells are increased in volume. × 130

FIG. 10.—Section from the centre of protected area of the same tumour (Case I), taken at the same time as section shown in Fig. 9. Radiation dose is 400 r. The histological picture is similar to that in Fig. 9 except that the fibrosis is somewhat less and the inflammatory reaction shows an earlier phase. × 130.

FIG. 11.—Section from the exposed area 18 days after 5000 r direct radiation. It received 280 r scatter radiation during this period. The connective-tissue stroma is loose, and there is little fibrosis. Polymorphonuclear leucocytes are invading into the tumour cord, around which there is an accumulation of lymphocytes, macrophages and plasma cells, indicating an inflammatory process. All tumour cells in the cord have undergone degeneration. × 130.

FIG. 12.—Section from the area adjacent to that shown in Fig. 11. It received 3500 r direct and 400 r scatter radiation. The radiation-induced changes in the tumour cord are similar to those seen in Fig. 11. Connective tissue of the stroma is loose, and displays a somewhat smaller inflammatory reaction than seen in Fig. 11. × 130.

1

2

3

4

Jolles and Koller.

9

10

11

12

fractionated dose, giving a total of 2800 r (Case II), all the cells in mitosis exhibit chromosome fragmentation, i.e. direct radiation injury ; on the other hand, in the adjacent protected regions (240 r scatter radiation) no abnormalities were seen in dividing cells (Fig. 7, 8). The absence of chromosome fragmentation is another proof that the alteration seen in the protected areas, such as the increase in the size of tumour cells, and the slight degree of fibrosis and inflammatory reaction, must be considered as an indirect reaction to radiation.

Our investigation has shown that the rate and degree of the indirect radiation reaction depends on the histological organization of stroma, and on the dose. On account of the differences in the histological architecture of tumours, the minimum effective dose varies from tumour to tumour. We found that in tumours in which the connective tissue is abundant the minimum effective dose is smaller than in tumours with scanty stroma. This is illustrated in Case II, in which the dosage used (2700 r actual dose in 35 days) did not reach the critical minimum level necessary to produce a reaction of sufficient strength and efficiency in the scanty connective tissue of the " protected " tumour areas. On the other hand, the use of the sieve-technique has permitted the subsequent treatment of the residual tumour with a relatively high but adequate dose (4500 r actual dose in 18 days), because the histological texture of the skin, owing to the small size of the areas directly exposed to irradiation during the initial treatment has been left unimpaired, and could safely be subjected to such a high dose in the second treatment.

The complexity of the various cellular and tissue interactions increases when further radiation is carried out through another " chess-board or sieve " in which the position of transparent and opaque areas is reversed. By increasing the

TABLE I.—*The Effect of Radiation on Cells and Tissues.*

| Cells and tissues. | *Intracellular effects.* Dividing cells. | *Intracellular effects.* Interphase and differentiated cells. | *Intercellular or tissue effects.* |
|---|---|---|---|
| Malignant | Chromosome fragmentation<br>Chromosome bridges<br>Nuclear deficiency<br>" Pycnosis "—cell death | Increase in cell size : "over-differentiation"<br>Disturbance in enzyme activity | Integrated effects—<br>Synchronous division of injured cells.<br>Regional breakdown and tissue necrosis.<br>Alteration in histological organization.<br>Undefined " physiological " effects with changes in surface tension, in pH, in osmotic pressure, etc. |
| Normal (tumour bed and stroma) | Same as above. Difference is only in degree and rate, not in quality of the induced effects | | Integrated effects—<br>*Stroma-reaction :* (i) *directly induced.*<br>(local defence-reaction).<br>Introduction or increase of the process of " inflammation."<br>Invasion of lymphocytes into connective tissue.<br>Plasma-cell differentiation.<br>Fibrosis ; excessive deposition of collaeng.<br>(ii) *Indirectly induced.*<br>Same as above, produced by "diffusible substances " in adjacent, non-irradiated areas. |

number of cycles, i.e. by alternating the chess-boards twice, three or four times the rate and intensity of the tissue reactions can be greatly affected. We found in the cases investigated that at the completion of treatment the excessive tissue fibrosis was absent, and that the tumour-bed and stroma reactions are uniform in degrees and type in regions which received significantly different doses, such as 5280 r and 3900 r in Case I (Fig. 11, 12) and 4560 r and 3610 r in Case II respectively. Furthermore it was observed that the histological alterations in the tumour-bed have brought about an environment which is particularly well suited for the development and maintenance of the " inflammatory " reaction, induced by the local defence system of the body under the influence of radiation.

The application of the sieve-method has enabled us to distinguish clearly between the various reactions in normal and malignant tissues, to study their integration and to estimate their relative importance in the radiation response of tumours (Table I, Jolles, 1949*b*). It became firmly established in the course of our investigation that the radiation reactions in the connective tissue of the tumour-bed and the stroma play a very important role in the destruction of tumours. We found that while the connective tissue needs protection from excessive radiation, it requires a certain quantity of radiation and a specific distribution in time and space of this quantity in order to elaborate the radiation-induced reaction. We have seen that on the one hand the sieve method greatly favours the induction and maintenance of this reaction, and on the other hand, it does not impair the basic structure of the histological organization of normal tissues.

### DISCUSSION.

In the present-day practice of radiotherapy of squamous epitheliomata, the most important guiding principle seems to be " the delivery of the highest dose possible within the shortest time," the effective dose being that which the skin can tolerate, and the aim is to suppress mitosis and induce " differentiation " in the tumour. The biological aspect which underlies the above concept takes into consideration changes or reactions within *individual* tumour cells, but disregards the tissue environment with its great variability (Scarff, 1948). This drastic simplification of the biological complexity of tumour behaviour is responsible for the establishment of some uniformity of treatment for the most diverse neoplastic growths.

The cytological study of the radiation reactions of tumours during and after treatment has shown that by the daily repetition of fractionated doses of 500–600 r, the reactions are accelerated and intensified to such an extent and in such a manner that the interaction of the various responses becomes greatly disorganized (Koller and Smithers, 1946 ; Koller, 1948). Extensive coarse fibrosis is of common occurrence, and may be produced in the stroma early in treatment. This not only prevents the development of an inflammatory process in the connective tissue of the tumour-bed, but a protective barrier is formed which shelters islands of tumour cells from further radiation damage. It has been found that the daily repetition of the fractionated dose more often than not interferes unfavourably with the tumour-bed and stroma reactions and greatly hinders their efficiency.

Since the fact that reactions of the connective tissue play an important role in the destruction of tumours has been disregarded in order to achieve " cures,"

the highest dose which the skin can tolerate is used. The stroma and tumour-bed reactions, however, being too obvious to disregard completely, are referred to, but only as concomitant, " independent " events, which have no, or too little, bearing on the radiation response of the malignant growth itself. The most cursory histological study of tumours under radiation reveals the fact that the destruction of all malignant cells of the tumour parenchyma rarely, if ever, can be achieved by the " direct " action of a therapeutic dose. It seems, therefore, necessary and logical not only to admit the existence of an " indirect " effect, but to accept all the implications of the generic statement that the purpose of radiation is to kill a variable but significant proportion of active tumour cells, and at the same time to assist the local body defence system by inducing the various responses in the tumour-bed and stroma.

It also follows that any view based on changes induced by radiation in some and not all tumour-tissue components is not comprehensive, and that the conclusions drawn from the study of cell behaviour alone are doubtful. On that account the "cell count method" of Glücksmann and co-workers (Glücksmann, 1941, 1948; Glücksmann and Spear, 1945; Glücksmann and Way, 1948), which *per se* represents a contribution to radiobiological knowledge, is liable to be misleading when claims are being attached to it, such as that it provides an explanation for the variation of radiation response of tumours, predicts the outcome of treatment, and indicates the most appropriate treatment method, whether surgical or radiological, for a given case. The cell count method is biased by subjective selection and interpretation; it disregards the great regional heterogeneity shown by tumours both in growth rate and histological organization, and the fact that a malignant growth is composed of tumour and stroma, which form one closely integrated unit. It is more than obvious that for that reason the application of the cell-count method to biopsy specimens taken after a " test dose " of radiation in order to decide whether a tumour be submitted for surgery or radiotherapy is of doubtful value (Glücksmann and Way, 1948).

It has been stated by Spear (1946) that " radiation affects any given cell of a complex tissue in at least two ways, first by direct action on the cell, and secondly by injuring neighbouring tissues, upon the healthy functioning of which the cell depends." Our investigation leads us to a similar conclusion that the direct cellular effects, e.g. fragmentation of chromosomes, represent only the initial phase, and that the most important factors responsible for the disorganization, regression and re-absorption of tumours during and after irradiation are the various, closely integrated and related reactions which are produced by the radiation in the connective tissue of tumour-bed and tumour stroma.

The present investigation, in which the alternating chess-board method was employed, has shown the role of the connective-tissue reaction in the destruction of tumours and the way by which this reaction can be favourably influenced and modified. We have found that the fractionation of dose in time as well as in space, as carried out by the sieve, induces specific changes in cells and tissues. The intensity and rate of these changes affects the whole architecture of stroma and tumour-bed as well as that of the tumour. Our investigation has also shown that when the sieve is used sufficient response can be produced in the local defence system of connective tissue, and can be maintained by a much smaller dose than usually employed. The protection of connective tissue by the sieve enables us in cases where the tumour is radio-resistant, either by its nature or by its environment, to deliver a higher total tumour-dose without the danger of destroying vital structures of the tumour-bed. It is our intention to investigate the potentialities of this new procedure by applying the sieve method for the treatment of radio-resistant and deep-seated tumours, especially metastatic deposits in lymph nodes. The sieve or chess-board method is not designed for the treatment of small superficial epitheliomata, which can be dealt with successfully by more expeditious techniques at present in use. It offers a standby in difficult extensive cases in which a real risk of necrosis has to be faced in order to eradicate the tumour, or in cases in which radiotherapy registers failures more often than cures.

### SUMMARY.

The radiation reaction in 18 cases of accessible tumours treated by the sieve-method has been analysed. Treatment has been carried out through a lead "sieve" or "chess-board" with square or circular apertures of 1·0–2·0 cm. in diameter. The tumour is divided into a number of "exposed" and "protected" areas, the former receiving direct, the latter receiving only a small amount of scatter and transmitted radiation. Two chess-boards or sieves, which differ in the order of the transparent (exposed) and opaque (protected) squares, can be used during treatment.

Irradiation induces injuries in the tumour cells and alterations in the connective tissue of the tumour-bed and stroma. The latter consists of fibrosis and a reaction closely resembling inflammation. The radiation reaction in the connective tissue plays a very important role in the destruction and reabsorption of tumours.

In tumours irradiated through the sieve it was observed that tissue-reaction is reduced in the directly "exposed" regions, but at the same time, fibrosis and inflammatory reactions are induced in the "protected" regions. No cellular injuries, due to direct radiation such as chromosome fragmentation, were seen in cells of the "protected" areas.

By using two sieves which differ in the order of the "transparent" and "opaque" areas, the reaction of the tumour-bed and stroma in the presence of abundant connective tissue becomes uniform throughout the tumour, irrespective of the different doses which regions may have received during treatment.

Analysis of the radiation reaction in tumours treated by the sieve has shown that fractionation of dose in time as well as in space induces injuries and specific alterations in the irradiated area, the rate and type of which greatly enhances tumour destruction.

The authors are indebted to the British Empire Cancer Campaign for a grant for technical assistance, and thanks are also due to Mr. F. E. Speed (Chester Beatty Research Institute) for the microphotographs, and to Mr. E. J. Pamely, Research Technician (General Hospital, Northampton).

### REFERENCES.

ANDERSON, R. S.—(1949) 'Investigations into Differentiation and other Morphological Changes in Malignant Tumours following Therapeutic Irradiation with X-rays and Radium.' Köbenhavn (Munksgaard).

BLOOM, W., AND JACOBSON, L. O.—(1948) *Blood*, 3, 586.

Caspari, W.—(1922) *Z. Krebsforsch.*, **19**, 74.
Czepa, A.—(1924) *Strahlentherapie*, **16**, 913.
Desjardins, A. V.—(1932) *Amer. J. Roentgenol.*, **28**, 398.
Ellis, F.—(1942) *Brit. J. Radiol.*, **15**, 348.
Elson, L. A., and Lamerton, L. F.—(1949) *Brit. J. Cancer*, **3**, 414.
Ewing, J.—(1926) *Amer. J. Roentgenol.*, **15**, 93.
Failla, G.—(1940) *Ibid.*, **44**, 649.
Friedman, M.—(1939) *Radiology*, **33**, 633.
Glücksmann, A.—(1941) *Brit. J. Radiol.*, **14**, 187.—(1948) *Ibid.*, **21**, 559.
*Idem* and Spear, F. G.—(1945) *Ibid.*, **18**, 313.
*Idem* and Way, S.—(1948) *J. Obstet. Gynaec.*, **55**, 573.
Harvey, W. F.—(1942) *Edinb. med. J.*, **49**, 529.
Jolles, B.—(1946) *Brit. J. Radiol.*, **19**, 220.—(1948) *Amer. J. Roentgenol.*, **60**, 745.—(1949*a*) *Lancet*, ii, 603.—(1949*b*) *Brit. J. Cancer*, **3**, 27.—(1949*c*) *Nature*, **164**, 63.—(1950) *Brit. J. Radiol.*, **23**, 265, 18.
Kok, F., and Vorlaender, K.—(1922) *Strahlentherapie*, **14**, 497.
Koller, P. C.—(1948) *Brit. J. Radiol.*, *Suppl. No.* 1, p. 84.
*Idem* and Smithers, D. W.—(1946) *Ibid.*, **19**, 89.
Lasnitzki, I.—(1947) *Ibid.*, **20**, 232.
Murphy, J. B., Maisin, J., and Sturm, E.—(1923) *J. exp. Med.*, **38**, 645.
Pohle, E. A., Ritchie, G., and Moir, W. W.—(1949) *Radiology*, **52**, 707.
Roussy, G.—(1926) *Cancer Brux.*, *Suppl. No.* 110.
Russ, S., Chambers, H., and Scott, G. M.—(1921) *Proc. Roy. Soc.*, **92**, 125.
Scarff, R. W.—(1948) *Brit. J. Radiol.*, **21**, 594.
Souttar, H. S.—(1929) 'Radium and its Surgical Application.' London (W. Heinemann, Ltd.)
Sugiura, K., and Cohen, J.—(1939) *Radiology*, **32**, 71.
Spear, F. G.—(1946) *Brit. med. Bull.*, **4**, 2.
Vernoni, G.—(1948) *Recenti progressi in Medicina*, **4**, 177.
Warren, S., and Dixon, F. J.—(1949) *Radiology*, **52**, 714.
Willis, R. A.—(1948) 'Pathology of Tumours.' London (Butterworth & Co., Ltd.)
Windeyer, B. W.—(1942) *Brit. J. Radiol.*, **15**, 236.
Windholz, F.—(1947) *Radiology*, **48**, 274.